# A Rapid Method to Achieve Aero-Engine Blade Form Detection

**DOI:** 10.3390/s150612782

**Published:** 2015-06-01

**Authors:** Bin Sun, Bing Li

**Affiliations:** State Key Laboratory for Manufacturing Systems Engineering, Xi’an Jiaotong University, Xi’an 710049, China; E-Mail: sun075634@stu.xjtu.edu.cn

**Keywords:** blade, laser displacement sensor, freeform surface, error compensation, four-coordinate measuring system

## Abstract

This paper proposes a rapid method to detect aero-engine blade form, according to the characteristics of an aero-engine blade surface. This method first deduces an inclination error model in free-form surface measurements based on the non-contact laser triangulation principle. Then a four-coordinate measuring system was independently developed, a special fixture was designed according to the blade shape features, and a fast measurement of the blade features path was planned. Finally, by using the inclination error model for correction of acquired data, the measurement error that was caused by tilt form is compensated. As a result the measurement accuracy of the Laser Displacement Sensor was less than 10 μm. After the experimental verification, this method makes full use of optical non-contact measurement fast speed, high precision and wide measuring range of features. Using a standard gauge block as a measurement reference, the coordinate system conversion data is simple and practical. It not only improves the measurement accuracy of the blade surface, but also its measurement efficiency. Therefore, this method increases the value of the measurement of complex surfaces.

## 1. Introduction

The blade is a key component of the aero-engine, but it can suffer many poor working conditions [1]. The blade surface is a complex three-dimensional surface, with a strong twist. The technical characteristics are of thin-walled parts and deformation. It has many high precision parameters, from the tip to the root of each section of the line. The measurement of the blade involves a three-dimensional curved surface measurement of space. Its complexity and diversity causes considerable difficulty in this measurement [2]. As one of the core components in aero-engines, when the detection accuracy of the blade is improved and the process quality is controlled, engine performance and service life can be effectively improved. In addition, when the blade works under high pressure and high temperature for a long time, it is prone to folding, twisting, dislocation, wear, corrosion, and other various defects. Once there is a blade failure phenomenon, it will seriously affect flight safety. The blade is expensive to manufacture. Regular blade repair service and testing not only extends the engine life, but also enhances economic efficiency and saves costs. Therefore, blade surface detection is of great significance to the maintenance of engine parts. In order to control the quality of the blade processing or repair, a quick and efficient detection of its geometric deformation has become the frontier field of advanced manufacturing needs to settle one of the most difficult problems.

The blade measurement methods include contact and non-contact measurements. The contact measurement methods include inductance measurements, standard model measurements, and CMM measurements [3,4]. CMM measurements can get the point cloud data; however they do not meet all the needs of obtaining three-dimensional information. Additionally the measurement speed of contact measurement methods is very slow. Optical non-contact measurements are widely used methods [5,6,7], and include the triangulation, fringe projection and stereovision methods. Although three-dimensional blade profile measurement using non-contact measurement methods has reached a certain level of sophistication, the accuracy and speed can still be improved [8,9,10].

Many researchers have directed their effort to solve these problems by trying to determine the number of points needed to control the path and the movements of the probe on the measured surfaces. Nishikawa and Ohno [11] developed a non-contact type of on-machine measurement system. This system enables one to measure glossy metal surfaces by employing the latest laser displacement sensor. Zhang [12] proposed a feature-based inspection process planning system for co-ordinate measuring machines (CMM). Hsu [13] described a complete procedure of blade measurement flow and analysis technique. A two-step measurement procedure, including a rough measurement and a fine measurement, along with the calculation of the back-off directions was proposed to improve the measurement accuracy. Lin *et al*. [14] proposed an automated setup sequence of points to be measured, categorizing the verifying data using distances and angles. Then, using a NUBS mathematical model fitted to smooth surfaces.

In this paper, according to the conditions and purpose of blade surface measurement, a rapid method to achieve aero-engine blade surface detection is proposed on the basis of previous studies. This method uses a non-contact Laser Displacement Sensor (LDS) to acquire the blade surface data. A new measuring device was developed and a new measurement path is presented. It can achieve better measurement speed and precision.

## 2. The Blade Measurement System

### 2.1. Laser Displacement Sensor

In recent years, laser measurement technology has developed rapidly as a representative non-contact method. Among them, the point laser non-contact measuring method based on the theory of laser triangulation, has a simple structure without applying force during measurement, high measurement accuracy, large measurement range and high efficiency, *etc*. It has been widely used in reverse engineering and rapid measurement of complex surfaces.

**Figure 1:**
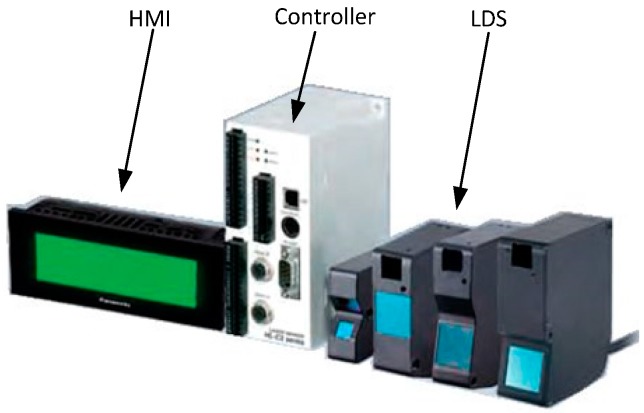
Laser Displacement Sensor (LDS).

The employed Laser Displacement Sensor LDS), shown in Figure 1, consists of a HMI, a controller and a HL-C211BE LDS, which are all produced by Panasonic (Aichi-ken, Japan). The LDS uses a red (658 nm) semiconductor laser as the light source. Its integrated circuit chip is a patented technology system. The LDS can work as a high-density light receiving element and close to the limit of the processing speed, so that it can achieve higher speed and resolution. The specific parameters are listed in Table 1.

The LDS’s controller has many functions. Compensation and parameters such as measurement methods, color of the objects, materials and roughness in the LDS have been optimized. When used according to the measured object characteristics through the operation of a panel dialogue menu, setting the appropriate parameters would reduce the error in order to improve the accuracy of detection of complex surfaces. In this paper, some experiments that were not performed by the manufacturer were made to compensate for inclination angle error.

**Table 1:** The main parameters of the LDS.

Names	Specifications
Model No.	HL-C211BE
Installation Mode	Diffuse Reflection
Measurement center distance	110 mm
Measuring range	±15 mm
Linearity	±0.03% F.S.
Beam diameter	80 μm
Repeatability	0.1 μm
Resolution	0.25 um
Temperature characteristics	0.01% F.S./°C

### 2.2. Measurement Mechanism Features and Parameters 

**Figure 2:**
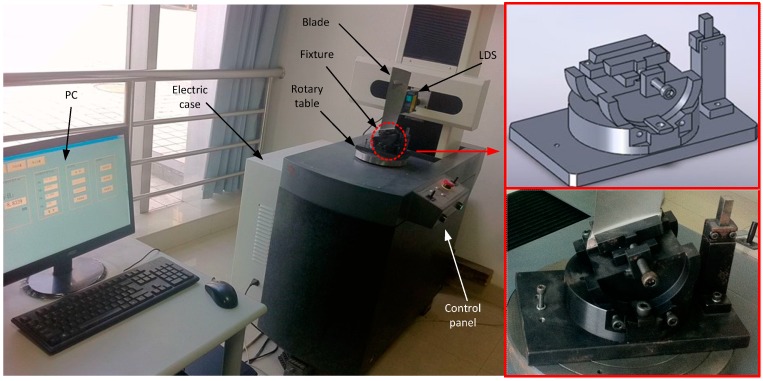
The blade measuring instrument and the fixture.

In view of the blade surface characteristics using non-contact laser measurement, a four coordinate measuring machine cantilever structure layout was designed: the four-coordinate measuring body includes three vertical coordinates (X-axis, Y-axis, Z-axis), and a rotary table. Each axis is operated by the servo motor control and movement along the a precision ball track. The rotary table is used to reduce the length of the cantilever, and to stabilize the cantilever structure from deforming. In the measurement, the main motion is from the rotary table movement and the movement of small displacement of the cantilever. In the improved cantilever type measuring system, the structure is simple, stable and reliable, and X- Y- Z axis load is light. The shaft has precision ball screw guided movement, overall system accuracy is less than 2 μm the equipment can meet the requirements of the production site blade surface testing. The measurement system architecture design layout is shown in Figure 2, and the coordinate axis parameters are shown in Table 2.

**Table 2:** Coordinate axis parameter list.

Name	X	Y	Z	Rotary Table (W)
Measuring range	220 mm	180 mm	360 mm	360°
Velocity	8~15 mm/s	8~15 mm/s	8~15 mm/s	3~15 Rev/min
Straightness	0.6 μm	0.4 μm	0.6 μm	Rotation 0.3 μm
Axis Misalignment	6"/200 mm	7"/200 mm	6"/200 mm	Runout ≤ 0.5 μm
Perpendicularity	1.6 μm	1 μm	1.8 μm	0.5 μm
Overall accuracy	≤2 μm			
Carrying	≥50 Kg			

**Figure 3:**
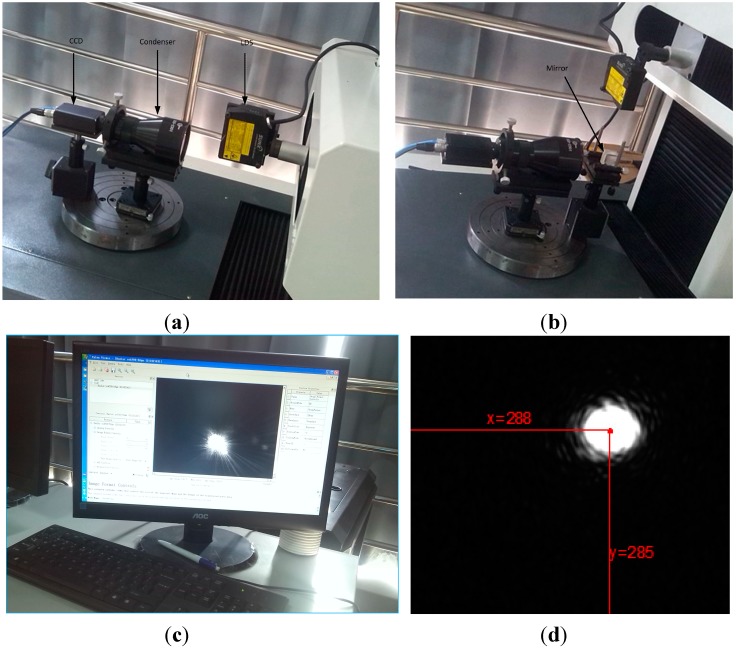
The LDS attitude adjustment. (**a**) Beam horizontal alignment; (**b**) Beam vertical alignment; (**c**) CCD acquisition display; (**d**) Light spot centroid coordinates.

As shown in Figure 2, the LDS was mounted in the X-axis of a four-coordinate measuring system. The blade is vertically arranged on the fixture, placed on the rotating platform W. Before measurement, the attitude of the LDS should be adjusted. As shown in Figure 3, it is necessary to carry out the calibration of the LDS beam to guarantee the accuracy. It only needs to ensure the direction of the beam and the axis in alignment to satisfy the Abel principle because the condition of X, Y, and Z axis has been determined by the mechanical structure. To display the location of the beam, a high precision scA780-54gm CCD (Basler, Ahrensburg, Germany) is selected.

The calibration principle of the horizontal beam is the same to the vertical beam, the principle of which is shown in Figure 3b. A 45° mirror to change the direction of the beam is needed. We move the location of the LDS along the axis and record the spots of the CCD when it stands in the two endpoints. As shown in Figure 3d, the coordinates of the light beam is obtained by using MATLAB to process the CCD images. The LDS fixture is slightly adjusted referring to the departure position to make sure the location of the light spot does not move. Finally the direction of the beam and the measuring direction of the LDS are confirmed as the same through repeated calibration.

Afterward, the attitude of the blade should be adjusted through the fixture. A special fixture was designed to measure the blade: It cannot only clamp various types of blades, but can also adjust the angle according to the situation of blade root tilt. The blade axis is consistent with the Z-axis. When adjusting the blade attitude, the central axis of the blade can been found out through the CMM first. Then the blade is mounted on the fixture and is placed approximately vertical on a rotating platform. By controlling the LDS along the Z-axis and fine-tuning the fixture attitude, it makes the laser point trajectory and the blade axis coincide. The result of the posture adjustment is a precision fine-tuning process. It usually requires many repetitions to complete. When measuring, the LDS determines the measured position of the blade by controlling each axis’ servomotor action. A PC program records the LDS values and Renishaw grating rulers mounted each shaft, so the coordinate position of each measurement point can be accurately positioned, and the purpose of accurate measurement will be achieved.

### 2.3. Control System Principles

**Figure 4:**
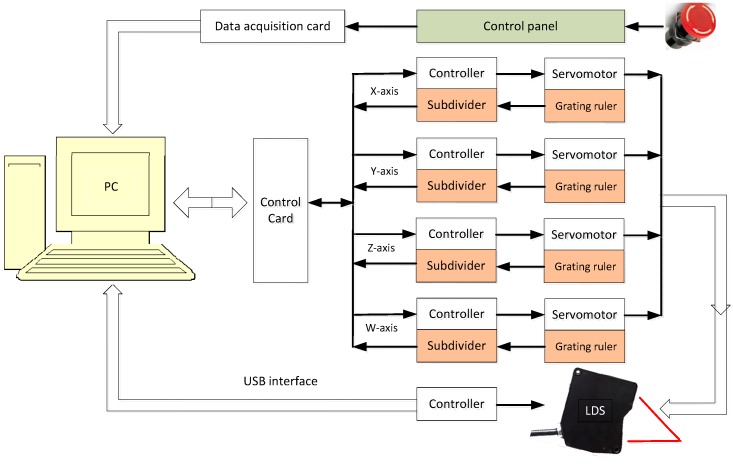
Measurement system control schematic.

The control system is one of the key components of the measuring equipment. The principle is shown in Figure 4. Its main functions are: PC software measuring instrument control of the X-axis, Y-axis, Z-axis linear motion and W-axis rotary turntable movement; then reading the spatial coordinate values through variety of movements to cooperate with each other; finally the blade surface measurement will be realized. The control system adopts a closed-loop feedback control. The measuring machine state is monitoring in real-time. Among them, the position of each axis movement is set by grating readout and on both ends of each axis a limit switch can prevent motor movement to the limit position, so the safety and reliability of the whole system is fully protected. Visual measurement and control software is compiled in Visual C++ for ease of operation and powerful functionality. The software is a modular design based on the characteristics of the blade. By using object-oriented thinking and combining powerful 3D OpenGL graphics capabilities, a special blade surface measurement system is developed. The measurement software can realize motion control of the devices, data acquisition and processing, the blade surface three-dimensional display, test results of statistical analysis, and other functions.

## 3. Laser Triangulation Principle and Inclination Error Compensation Model

### 3.1. Laser Triangulation Principle 

The LDS is a designed precision optical instrument based on the principle of laser triangulation. It can transform displacement information of optical signals into electrical signals by the laser triangulation method, which measures the triangle formed by the incident beam and the reflected laser beam. The measurement principle is described as follows: the laser transmitter axis, the optical axis of the lens acceptance and the charge-coupled device (CCD) linear array are all in the same plane; the laser emits a beam of parallel light, focusing on the object surface by the condenser lens; then, the laser beam is received by an imaging lens at another angle due to the diffuse reflection and a spot is formed on the sensitive surface of the CCD linear array sensor of the LDS; if the position of the laser point changes because of the displacement of the changing measured object, the position of the spot on the CCD linear array sensor of the LDS changes as well; the position of the laser point *x* on the surface of the measured object can be calculated according to the position of the spot *x′*.

**Figure 5:**
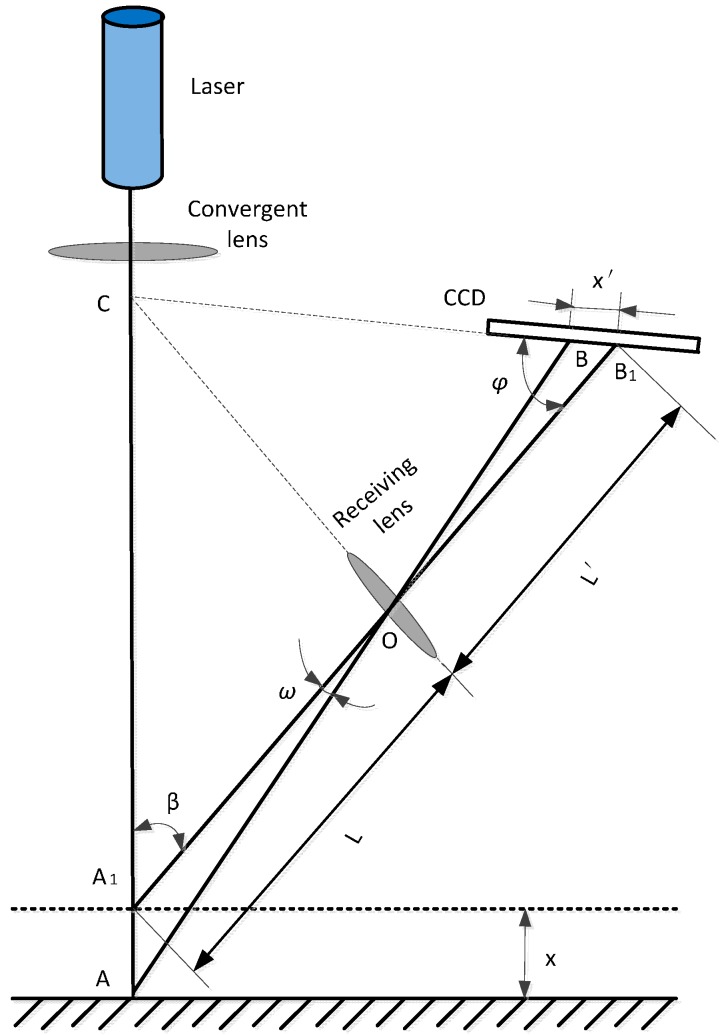
The schematic diagram of direct laser triangulation.

Figure 5 shows a typical direct laser triangulation schematic; an optical system consists of a laser transmitter, convergent lens, receiving lens, the CCD linear array sensor and subsequent signal processing circuit, etc. Suppose the light spot emitted by the LDS irradiates on the surface of the measured object position A, Point B is a reflective spot on the CCD imaging position. If the position of point A_1_ changes and movement distance is *x*, thus the displacement of the spot on the CCD image point is *x′*. 

The trigonometric sine theorem is as follows:

(1) $$x=\frac{Lx^{\prime }sin\text{φ}}{L^{\prime }sin\text{β}-x^{\prime }sin\left(\text{φ}+\text{β}\right)}$$

In the above formula, *x* is the movement distance of the object and *x′* is the movement distance of corresponding image point; *ω* is the angle between the two beams of reflection; β is the angle between the incident beam and the axis of the receiving lens; φ is the angle between the surface of the photosensitive CCD receiving lens axis; *L′* is the object distance of receiving lens, which is the distance between point A and receiver lens; *L′* is the image distance of receiving lens, which is the distance between receiver lens and the center of the imaging surface.

Because the internal light path of the LDS should satisfy the Scheimpflug condition [15], the imaging line of the CCD, the plane of the imaging lens and the laser beam meet at the point C. The relative position of each measured object point, the imaging lens and the CCD satisfy the Gauss theorem [16]. Then the measured object point can be imaged on its conjugate imaging plane, it ensures the measuring accuracy of the LDS. The Scheimpflug condition is shown as: *Ltan* φ *= L′ tan* β*.*

### 3.2. Inclination Error Model

According to the measurement strategy described in Section 3.1, the parameter of the LDS design is based on incident light being perpendicular to the surface. Once the incident light is inclined, it will produce an inclination error. This is because the inclination of light scattering from the lens changes the spatial distribution of the reception, that the position of the converged spot on the CCD light centroid is changed. If the calibrated model is still used to calculate the amount of displacement, it will produce errors. This is the root cause when the object plane is inclined to produce a measurement error. Research shows that compensating the inclination angle error of the LDS can effectively improve the accuracy of the measurement surface. The quantitative variation of the incident angle (or inclination angle) error is analyzed according to the relevant literature. 

**Figure 6:**
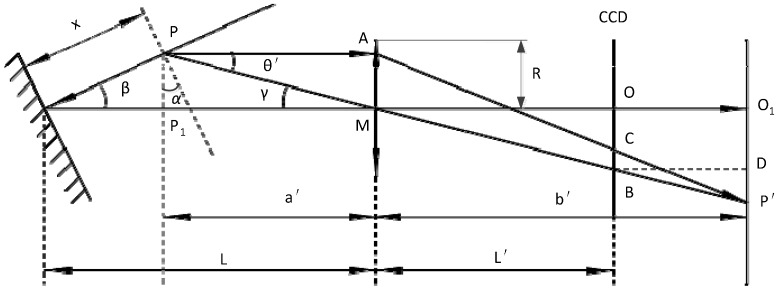
Schematic of the imaging performed by the receiving lens.

By the optical imaging principle shown in Figure 6, we can get quantitative model inclination error, which can be expressed as [17]:

(2) $$E_{\text{α}}=\frac{R^{2}L^{\prime }xcos\text{β}}{L^{3}}\left(1+2\frac{x}{L}cos\text{β}\right)\left[tan\text{β}-tan\left(\text{β}-\text{α}\right)\right]$$

Figure 6 shows a schematic of the imaging performed by the receiving lens. *P* is the measurement point; *a’* and *b’* are the object distance and image distance; *x* is the distance between *P* and the reference plane; and *f* is the focal length of the receiving lens; ***PA*** is the light centroid line, which passes through the receiving lens and creates an image on the CCD; *P’* is the image of *P*. 

In the model above, the only two input variables are the incident angle α and displacement of the object. *R*,β*, L* and *L′* are design parameters of the LDS. These analysis lead to the following three conclusions:

1. When the object surface inclination α is constant, the measurement error *E_α_* increases with increasing depth of field measurements;
2. When the displacement *x* of the object is constant, the measurement error *E_α_* increases with increasing body surface angle increases;
3. When α>0, direction of the error is the same with the direction of displacement; When α<0, direction of the error is the same displacement in the opposite direction;

In the model above, the only two input variables are the incident angle α and the displacement of the object. It provides a good prediction of the error tendency and allows a reasonable level of error compensation. In spite of the assumptions mentioned above, from the perspective of an engineering application, the quantitative models can improve the precision of measurement. Thence, it has a good sense of value and promotion.

### 3.3. Inclination Error Compensation Experiment

**Figure 7:**
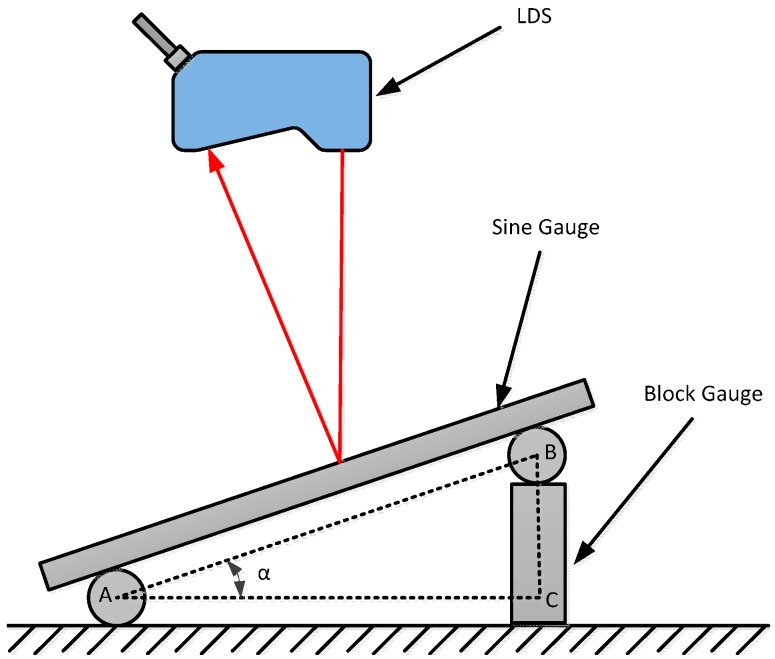
Schematic diagram of Inclination error experiments.

In order to verify the inclination error model derived in Section 3.2, the following experiments were done using a laser interferometer to test the LDS. Traceability of the laser wavelength characteristics of the Laser Interferometer (LI), and it has a very large measurement range and it can also reach nanometer measurement accuracy in a lab environment. Even considering the measurement uncertainty, it can also meet the needs of the project. It can achieve position detection for some large-scale and high-precision instruments, and in a number of precision measuring instruments or work piece calibration and correction. In this paper, the LDS inclination angle error compensation experiments use a Renishaw XL-80 Laser Interferometer as a calibration reference. The main parameters are as follow: Measuring range is 80 m; resolution of the HL-C211BE is 1 nm; linear accuracy is ±0.5 ppm. Inclination angle error experimental device of the LDS includes a four-coordinate measuring system, sine gauge, standard block gauge, laser displacement sensors and other components. The sine gauge is used as a measured object surface shown in Figure 7, where the sine gauge of the angle promotes a standard block gauge to adjust. It is calculated as follows:

In ∆ABC:

(3) BC=AB·sinα

Equation (3) shows the need to build a certain degree of inclination angle. The desired value can be obtained by calculating the amount of blocks.

**Figure 8:**
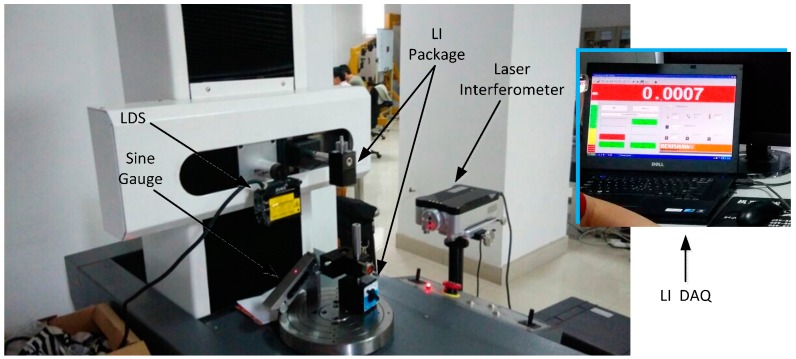
Inclination error experiments.

As shown in Figure 8, the LDS was mounted in the X-axis of a four-coordinate measuring system. The sine gauge was placed on the rotating platform that lied directed below the LDS. The Laser Interferometer optical module appurtenance was fixed by a magnetic force standing in the X-axis and rotary platform; the PC program controlled the X-axis movement along the Z-axis.

Before the start of the experiment, the precision laser sensor attitude is adjusted, which has been discussed in Section 2.2. The position of the laser interferometer optical components is adjusted to ensure the optical path shift does not shift in the process of moving down the Z-axis in order to guarantee accurate readings of the laser interferometer. Secondly, 5°, 16°, 28°, −6° angle sine gauge are built in the experiments. The sine gauge is placed just below the LDS. When the angle is positive, the inclination direction of the sine gauge corresponds to the sensor beam-receiving surface. Beginning of the experiment, a PC program moves the Z-axis up and brings down the LDS within the effective range of −15 mm~15 mm, recording the scale value of the LDS and laser interferometers every 1 mm, the LDS indication by the HMI, the value of the laser interferometer readout in the window which comes with the software through the PC.

**Figure 9:**
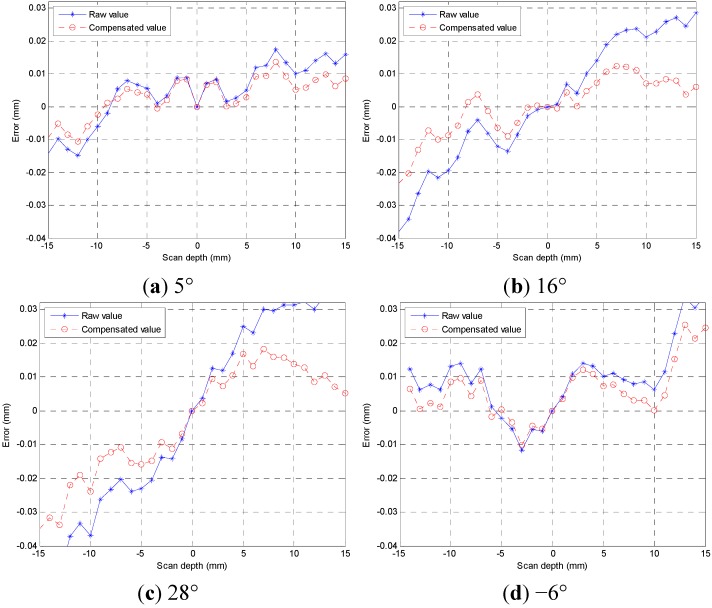
Inclination angle errors and compensation graph.

The experimental data is collated and can be compensated by the quantifiable error model in Section 2 of this paper. MATLAB processes and visually displays data in a two-dimensional pattern shown in Figure 9. The blue asterisk-dot curve represents the raw value error. The red round-dot curve with circles is compensated value error. It is clear that the error of the measurement data is effectively corrected, with error value within 10 μm. Accuracy is improved significantly.

## 4. The Blade Surface Measurement Path Planning

### 4.1. Measurement Path Constraint Condition

**Figure 10:**
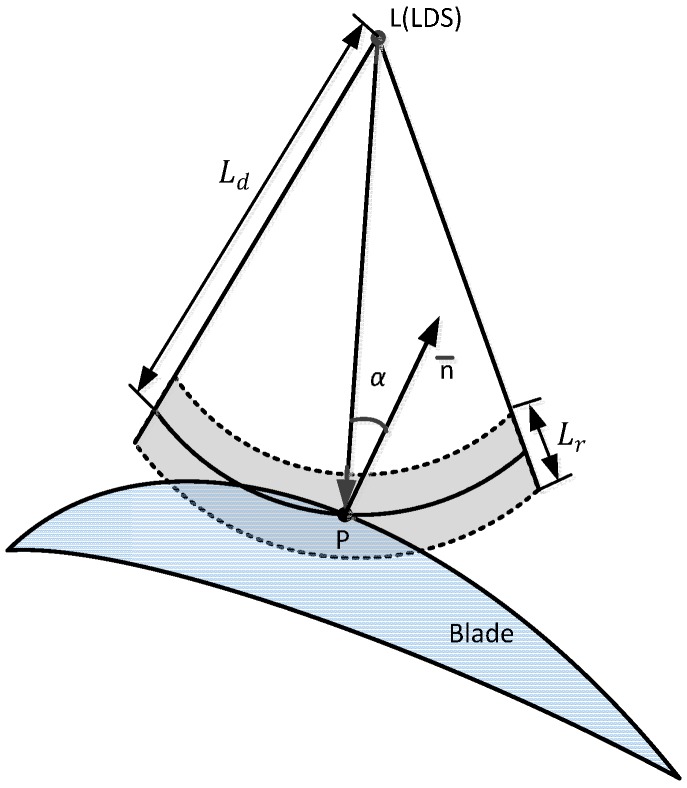
Schematic diagram of the LDS constraints.

The blade surface is irregular and the distortion is large. To ensure that the LDS works in the best position within the depth of field range in the process of blade profile measurement, the measurement path must be planned. Measurement path planning objectives are based on the measurement point and the LDS value information, planning the sensor movement trajectory, and meeting the job requirements of the LDS. Then measurement path is obtained that would reflect the curvature changes of the blade. As shown in Figure 10, the following formula is established [18,19,20]:

(4) $$L_{d}-L_{r}/2\leq \left|LP\right|\leq L_{d}+L_{r}/2$$

In the expression above, $L_{d}$ is the measurement center distance of the LDS; $\;L_{r}$ is the measurement range of the LDS;  LP is the distance between the LDS to the measurement point. Wherein, $L_{d}$ and $L_{r}$ are known characteristic parameters of the LDS; $\left|LP\right|=\sqrt{{\left(x_{L}-x\right)}^{2}+{\left(y_{L}-y\right)}^{2}+{\left(z_{L}-z\right)}^{2}}$, $x_{L},\;y_{L}$ and $z_{L}$ are the coordinates of the center values of the LDS, x, y and z are the corresponding points’ coordinates on the blade surface.

### 4.2. The LDS Scanning Path Planning

**Figure 11:**
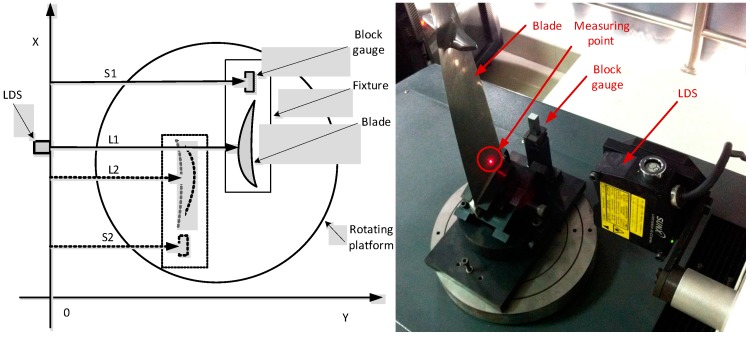
Measurement path planning diagram.

This paper adopts the direct type laser displacement sensor, as shown in Figure 11, in the measurement process, not only to ensure that the LDS is within the effective work range, but also to ensure that the measuring point inclination α = 0 by making the LDS incident ray and measurement points of the normal vector $\bar{\;n}$ coincide. The theory to measure the blade profile curve is unknown so in reality it is difficult to ensure that the normal incident beam and the measured point coincide. According to the characteristics of non-contact measurement, this paper presents a rapid method for establishing the blade measurement path planning which is suitable for engineering applications. In the two-dimensional plane of the blade features section, it will not consider inclination error first. The contour of one side is scanned by equal intervals to measure the constraint conditions. After the end of the data collection, inclination measurement points are calculated. The measuring point error can be calculated using inclination error quantitative model. Finally, after correcting the measured data, the actual measured value of the measuring point can be obtained. Similarly, the blade is rotated at 180° to acquire data along another edge. The blade section profile curve can be obtained after the data correction.

Scan path steps (see Figure 11):

Step 1: The measured blade is mounted on a special fixture after adjusting the blade profile to ensure that the blade stacking axis and Z-axis are parallel in measurement systems;

Step 2: When measuring, the X-axis positions are adjusted to ensure the standard gauge block falls within the measuring range of the LDS; the LDS collects data of the standard gauge block away one side S1; the rotary table was rotated 180°; another side of the standard gauge block data S2 is acquired;

Step 3: Adjust the LDS to the characteristics to be measured on the blade cross section, along the X-axis scanning the blade feature section; the data sequence of L1 is acquired by equal space; the solid lines are shown in Figure 10; Similarly, the rotary table was rotated 180°; the data sequence of L2 is acquired by equal space; the dotted lines are shown in Figure 10;

Step 4: The standard gauge block of dimension H is known; by the coordinate transformation, the characteristics of blade section data from two scanning can be converted under the same coordinate system; the characteristics of the cross section curve can be fitted out basing on these data; and then as needed, the characteristics of blade section parameters can be obtained;

Step 5: By moving the LDS along the Z-axis direction, different characteristic cross-sectional blade heights may be measured.

**Figure 12:**
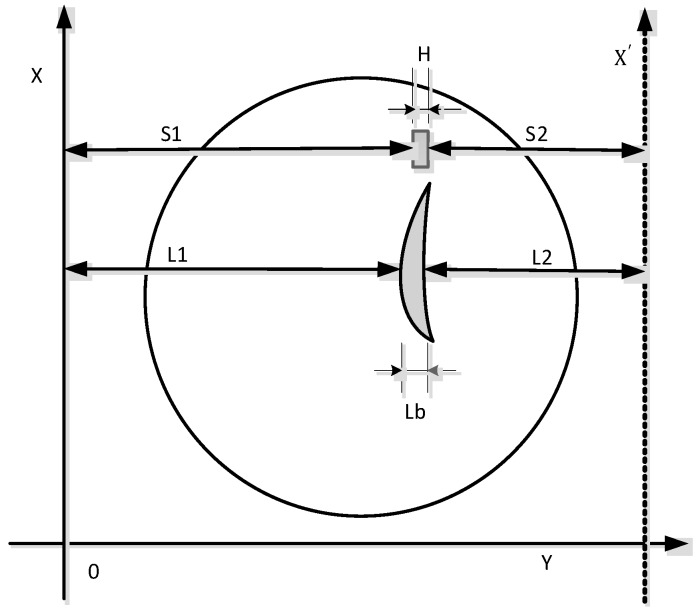
Measuring points sectional diagram calculation.

After the scan blade profile is complete, according to the geometric relationship shown in Figure 12, the data sequence L1, L2 is processed as follows:

(5) $$XX^{\prime }=S1+S2+H$$

Wherein the data sequence L1, L2 corresponding blade thickness:

(6) $$Lb=XX^{\prime }-L1-L2\;$$

Sequences L2 may be expressed as sequence L1:

(7) L2=L1+Lb

Here, H is the standard gauge block thickness; Lb is a sequence point $L1_{i},\;\;L2_{i}$ corresponding to the blade thickness, among them, i=1, 2, ···, n.

In the measurement process, to ensure that the measuring point in the measuring range of the LDS, coordinates Y need to be adjusted at any time. If the Y-axis mobiles distance Δy, so XX’ changes XX' + Δy, through the above formula can get the correct data.

The advantages of the blade section scanning path presented in this paper are: motion control is simple; data acquisition is fast; coordinate conversion algorithm is easy; engineering applications can be achieved. Although the path of the blade section considers the sensor measurement range constraints, inclination errors are inevitable due to non-uniform surface blade surface. In order to reduce or eliminate measurement errors, inclination of the measuring points need to be found. Appropriate error values may be calculated using the inclination on the error model, and then the raw data collected by LDS are corrected, the measurement accuracy can be effectively improved. The following calculation methods for the inclination of measuring points were discussed in detail.

### 4.3. The Calculation Method of the Blade Surface Inclination Angle

**Figure 13:**
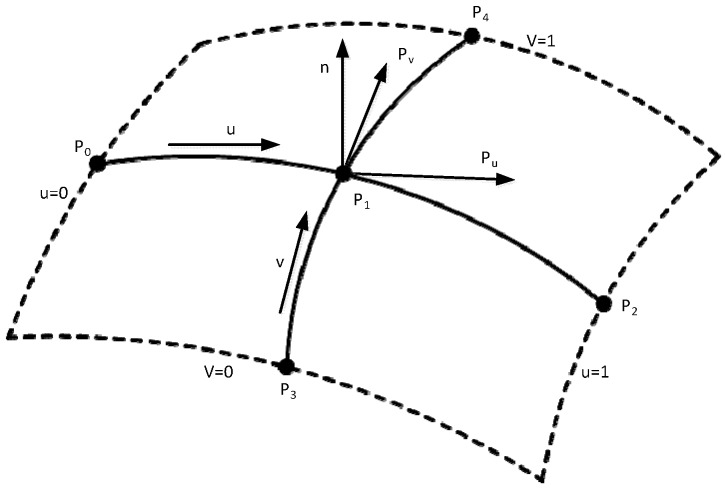
Quadratic Bezier curves intersect.

Before measuring the vane type blade, the inclination calculation method needs to be known. It is necessary to research the method of calculating the normal vector of the free surfaces measurement points. In this section, an improved cross-curve method [17,21,22] based on five data points is developed to calculate the unit normal vector. As shown in Figure 13, the LDS scans along two crossed curves at equal step size. One curve is longitudinal and the other is latitudinal. They intersect at the measurement point$\;P_{1}\left(x_{1},y_{1}\right)$. Four points $P_{0}\left(x_{0},y_{0}\right)$、$P_{1}\left(x_{1},y_{1}\right)$、$P_{2}\left(x_{2},y_{2}\right)$、$P_{3}\left(x_{3},y_{3}\right)$、$P_{4}\left(x_{4},y_{4}\right)$ on the two curves are chosen: Two on the latitudinal curve and the others on the longitudinal curve. $P_{u}$ and $P_{v}$ are tangent vectors, and n is the normal vector of $\;P_{1}$. In addition, the five points are arranged such that one central point is surrounded by four peripheral points approximately 90 degrees apart.

A quadratic Bezier curve (degree 2/order 3) that passes through the three points $\;P_{0}$,$\;\;P_{1}$ and$\;P_{2}$ can be expressed as [18]:

(8) $$P\left(u\right)={\left(1-u\right)}^{2}B_{0}+2u\left(1-u\right)B_{1}+u^{2}B_{2}$$

where $B_{0}$, $B_{1}\;$and $B_{2}$ are the control points, which are derived as $B_{0}=P_{0}$, $B_{2}=P_{2}\;$and $u=u_{1}$.

(9) $$B_{1}=\frac{-{\left(1-u_{1}\right)}^{2}P_{0}+P_{1}-u_{1}^{2}P_{2}}{2u_{1}\left(1-u_{1}\right)}$$

For the point $\;\;P_{1\;}$, tangent vector$\;P_{u}$ can be expressed as:

(10) $$P_{u}=P^{\prime }\left(u_{1}\right)\;=-2\left(1-u_{1}\right)P_{0}+2\left(1-2u_{1}\right)\times \frac{-{\left(1-u_{1}\right)}^{2}P_{0}+P_{1}-u_{1}^{2}P_{2}}{2u_{1}\left(1-u_{1}\right)}+2u_{1}P_{2}$$

where, the curve through three points $P_{0}\left(x_{0},y_{0}\right)$,$\;P_{1}\left(x_{1},y_{1}\right)\;$and $P_{2}\left(x_{2},y_{2}\right)$, the parameter $u_{1}\;$can be expressed as the formula:

(11) $$u_{1}=\frac{\sqrt{{\left(x_{1}-x_{0}\right)}^{2}+{\left(y_{1}-y_{0}\right)}^{2}+{\left(z_{1}-z_{0}\right)}^{2}}}{\sqrt{{\left(x_{1}-x_{0}\right)}^{2}+{\left(y_{1}-y_{0}\right)}^{2}+{\left(z_{1}-z_{0}\right)}^{2}}+\sqrt{{\left(x_{2}-x_{1}\right)}^{2}+{\left(y_{2}-y_{1}\right)}^{2}+{\left(z_{2}-z_{1}\right)}^{2}}}$$

Similarly, the other tangent vector $P_{v}$ at the central point of another curve can also be calculated as $P_{v}=P^{\prime }\left(v_{1}\right)$. Hence, the unit normal vector n at the central point $P_{1}\left(x_{1},y_{1}\right)$ is:

(12) $$n=\frac{P_{u}\times P_{v}}{\left|P_{u}\times P_{v}\right|}\;\;$$

Because the blade surfaces are non-uniform, there must be some inclination in measuring the LDS measuring point and the incident beam. Only when the right angle of the measuring point method about normal vector and the incident beam is found then the achieved data is accurately compensated. Measurement errors caused by the inclination angle are eliminated, so the blade surface accuracy is improved. In order for the algorithm to be implemented in engineering applications, blades should be mounted upright in the four coordinate system combining features of blade surface structure. As shown in Figure 13, the axial direction of blade profile should be kept parallel with the Z-axis to approximately coordinate the measuring system. The cross section line is perpendicular to the Z-axis, and measuring points are within the X-Y plane, so only the calculation method of tangent vector of measurement points on the curve need to be known.

**Figure 14:**
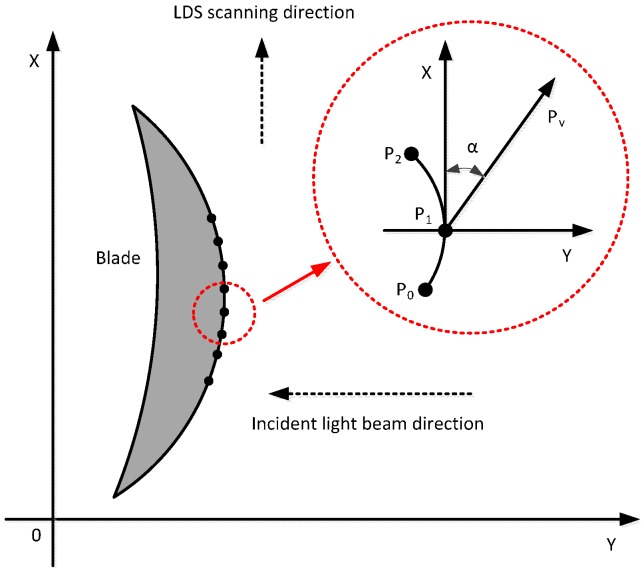
Planning and measuring point blade pitch angle calculation schematic.

By the geometric relationship shown in Figure 14, the tangent vector$\;P_{u}\left(x,y\right)$ of the measured point$\;P_{1}$ is known. If the tangent vector$\;P_{u}\left(x,y\right)$ of the measured point $\;P_{1}$ is parallel to the X-axis, then the incident light beam along the Y-axis is vertical to the vector $P_{u}$, and the angle of measuring points is equal to zero; otherwise the measuring point inclination α can be expressed as:

(13) $$\text{α}={tan}^{-1}\frac{y}{\sqrt{x^{2}+y^{2}}}\;\;$$

According to the coordinates of the neighboring point, when the tangent vector $P_{u}$ of the measuring point within the X-Y plane is known, the inclination of the measuring point can be calculated according to the above formula. Then the error value $E_{\text{α}}$ of the angle can be calculated from Equation (2), and raw measurement data $P_{o}$ is compensated, the exact value $P_{c}$ of the measuring point can be obtained:

(14) $$P_{c}=P_{o}-E_{\text{α}}$$

## 5. The Experimental Data Analysis

According to the experimental design, the measurement data of a characteristic cross-section of blades was analyzed. In the blade coordinate measuring system, the data is acquired by the LDS is one-dimension on the blade surface, so the measured value of the Y coordinate is equal to the value of LDS added to the value of the Y-axis grating ruler. According to the algorithm described in Section 4.3, the inclination angle of a measuring point can be calculated first, then put it on behalf of the error model and the error value is obtained. Finally, the data of the LDS can be corrected quite well. Data measurement process is shown in Table 3.

**Table 3:** Part of the measuring data compensation list (mm).

No.	X-Axis	RawL2	Angle	Error	Exact
…	…	…	…	…	…
220	88.7216	1.3578	12.1418	0.0025	1.3553
221	89.7509	1.4703	13.4518	0.0027	1.4676
222	90.5905	1.6135	14.1069	0.0030	1.6105
223	91.4285	1.7280	14.1837	0.0032	1.7248
224	92.4542	1.8773	14.3970	0.0034	1.8739
225	93.2782	2.0230	15.0744	0.0037	2.0193
226	94.1166	2.1383	16.3334	0.0039	2.1344
227	94.9440	2.2918	15.9000	0.0042	2.2876
228	95.9858	2.4555	16.1109	0.0046	2.4509
229	96.8228	2.6163	16.5751	0.0049	2.6114
230	97.6458	2.7550	17.0762	0.0051	2.7499
231	98.4832	2.9133	17.9380	0.0054	2.9079
232	99.5132	3.1010	17.8846	0.0058	3.0952
233	100.3537	3.2683	17.6309	0.0061	3.2622
234	101.1788	3.4335	18.4268	0.0065	3.4270
235	102.0151	3.5970	18.5351	0.0068	3.5902
236	103.0547	3.7943	19.1727	0.0072	3.7871
237	103.8791	3.9696	19.5836	0.0075	3.9621
238	104.7187	4.1545	19.7252	0.0079	4.1466
239	105.7483	4.3723	19.6610	0.0083	4.3640
240	106.5852	4.5598	19.7357	0.0087	4.5511
241	107.4090	4.7570	20.1276	0.0091	4.7479
242	108.2464	4.9363	19.6711	0.0095	4.9268
243	109.2880	5.1550	20.9436	0.0099	5.1451
244	110.1149	5.3635	21.2326	0.0104	5.3531
…	…	…	…	…	…

The blade used in experiments is from an aero-engine in service, its profile has been deformed and theoretical data was unavailable. In order to verify the reliability of the measurement results, the blade data was measured by the high-precision CMM. This CMM comes from Hexagon (North Kingstown, RI, USA) and the model was a Global classic SR575. Its measurement system uses a TESASTAR-m automatic indexing probe head and LSP-X1s continuous scanning probe, overall measurement accuracy ≤1.9 μm. Experiments show that when measuring the same five cross-section of the blade, the CMM takes 25 min. The method proposed in this paper only needs 11 min. It is clear that the proposed method has obvious advantages in measuring efficiency.

**Figure 15:**
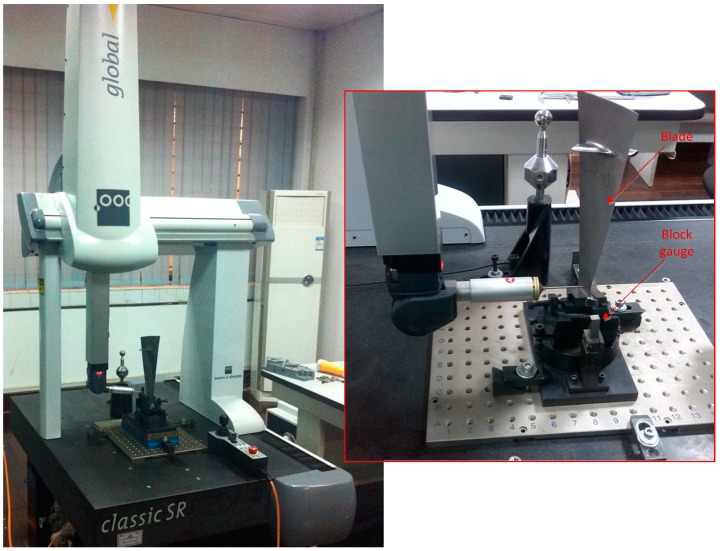
The CMM measuring the blade section features.

**Figure 16:**
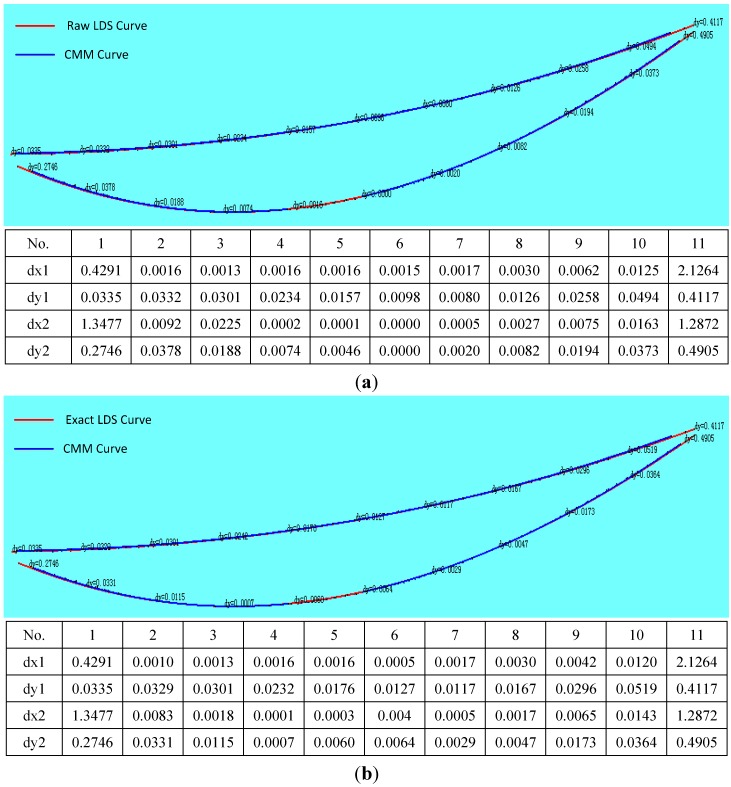
Curve deviation assessment. (**a**) Curve deviation assessment before compensation and data sheet; (**b**) Curve deviation assessment after compensation and data sheet.

The data measured of the same cross-section of the blade with CMM and LDS must be analysed to verify the correctness of the method. Before the measurement, a unified coordinate system is established for the blade to compare the test data because the blade surface is a complex surface, and coordinate values are also a complex issue. As shown in Figure 15, the blade is mounted the fixture, so two of the attitudes will never change, once they have been adjusted. According to the characteristics of the blade fixture, the surfaces of the outermost of the three standard gauge blocks are regarded as the CMM of the reference plane to establish the coordinate system. The edge of the blade end is greatly thin, the thickness of which is less than 40 μm. Due to the restrictions of structural features, CMM and LDS cannot achieve accurate detection of blade edges. Therefore only the area within 1 mm from the edge can be measured, and in this region, CMM and LDS are uniformly sampled along each aside 60 data points. Finally, using Imageware13.2 software to fit the data measured into graphs, and evaluating the curves deviation, as shown in Figure 16, it can be seen that the details of the compensated curve are close to the CMM data.

## 6. Conclusions

The blade is one of the most critical parts of an aero-engine. Because of its complex surface and difficult process, its profile form plays a decisive influence on the performance of the aero-engine. This paper presents a method of rapid realization to detect the aviation blade surface, which enables the collection of blade surface measurement data quickly and accurately.

First, the inclination error of the LDS is analyzed in the paper. From the principle of laser triangulation measurement, the geometry of the laser light path system is used in a detailed analysis and research. According to the mechanism in view of the inclination error, a quantified mathematical model is deduced. By using a special blade fixture and standard gauge block, a quick measurement path was planned out. After correcting the raw data with the inclination error model, the measurement precision of the laser displacement sensor is less than 10 μm. Finally, by the experiments comparing the LDS with a high-precision CMM, the LDS accuracy is significantly improved; measurement results have been significantly improved and enhanced.

The experimental results show that the rapid method presented in this paper achieves aero-engine blade surface detection. It can not only improve the measurement accuracy of the laser displacement sensor, but can also greatly improve the efficiency of the blade surface detection. This has great practical application value whether in protecting the quality of the blade at its production manufacturing site, or in controlling the quality of the blade after repair service.
